# A Chemically Induced *Vibrio harveyi* Bacterial Ghost Vaccine Confers Enhanced Protection in Turbot (*Scophthalmus maximus*)

**DOI:** 10.3390/vaccines14010109

**Published:** 2026-01-22

**Authors:** Haixiang Lv, Jianye Yang, Ruofan Yu, Qin Liu, Xiaohong Liu

**Affiliations:** 1 State Key Laboratory of Bioreactor Engineering, East China University of Science and Technology, Shanghai 200237, China; y30230453@mail.ecust.edu.cn (H.L.); y85240047@mail.ecust.edu.cn (J.Y.); y85220088@mail.ecust.edu.cn (R.Y.); qinliu@ecust.edu.cn (Q.L.); 2 Shanghai Engineering Research Center of Maricultured Animal Vaccines, Shanghai 200237, China; 3 Laboratory for Aquatic Animal Diseases of MOA, Shanghai 201400, China

**Keywords:** *Vibrio harveyi*, bacterial ghosts, turbot, inactivated vaccine

## Abstract

Background: *Vibrio harveyi* is a major bacterial pathogen threatening turbot aquaculture, necessitating the development of more effective vaccines. Bacterial ghosts (BGs), which are empty bacterial envelopes with preserved surface antigens, offer a promising alternative to traditional formaldehyde-killed vaccines that often suffer from reduced immunogenicity. Methods: We developed an optimized BGs vaccine for *V. harveyi* by combining the nonionic surfactant NP-40 with sodium hydroxide (NaOH). This NP-40/NaOH combination demonstrated a synergistic lytic effect, halving the minimum inhibitory concentration of NaOH required for complete inactivation. Results: The resulting BGs exhibited intact cellular morphology with transmembrane pores, efficient removal of cytoplasmic contents, and significantly better preservation of lipopolysaccharide structure compared to NaOH-alone treatment. Vaccination trials in turbot demonstrated that the NP-40/NaOH BGs provided the highest relative percent survival (RPS = 58.8%) upon challenge, outperforming both NaOH-alone BGs (RPS = 55.0%) and a traditional formaldehyde-killed vaccine (RPS = 34.8%). The superior protection was correlated with the induction of a more robust and sustained immune response, characterized by significantly higher levels of specific IgM antibodies, elevated lysozyme activity, and increased total serum protein. Conclusions: This study establishes the NP-40/NaOH protocol as an effective strategy for producing high-quality BGs with enhanced immunogenicity, presenting a potent vaccine candidate for controlling vibriosis in aquaculture.

## 1. Introduction

The turbot (*Scophthalmus maximus*) is a high-value economic species in Chinese mariculture, but its intensive cultivation is frequently threatened by infectious diseases, leading to substantial economic losses [1,2]. Among various pathogens, the Gram-negative bacterium *Vibrio harveyi* is a notorious marine pathogen with a broad host range, capable of infecting diverse vertebrates and invertebrates. It can invade farmed aquatic animals through multiple routes, including the skin, gills, and oral cavity [3]. Turbot exhibit symptoms such as ulceration and ascites after infection. When humans accidentally consume fish containing *V. harveyi*, it may lead to gastroenteritis, severe necrotizing soft tissue infections, and primary septicemia [4].

Vaccination has been established as one of the most effective strategies for controlling bacterial diseases in aquaculture. Among various vaccine types, inactivated vaccines are the most widely adopted in the industry due to their favorable biological safety profile, manufacturing stability, and ease of large-scale production. Traditional inactivated vaccines are typically prepared using chemical agents such as formaldehyde to neutralize pathogens. A historical example is the first commercially available aquatic vaccine, which was developed by inactivating *Aeromonas salmonicida* with formaldehyde [5]. However, a significant drawback of this conventional method is that the formaldehyde inactivation process can destroy or alter critical antigenic epitopes on the pathogen’s surface, leading to vaccines that exhibit insufficient immune persistence and diminished immunogenicity. Consequently, formaldehyde-inactivated vaccines frequently require the incorporation of multiple adjuvants to achieve a satisfactory level of protection, which adds complexity and cost to vaccine formulation [6].

Bacterial ghosts (BGs) represent a novel class of vaccine candidates, defined as empty but structurally intact bacterial cell envelopes generated through controlled lysis processes. The preparation involves creating precise pores in the cell membrane, enabling the selective expulsion of cytoplasmic components such as nucleic acids, ribosomes, and soluble proteins, while meticulously preserving the native architecture of the outer membrane and its associated surface antigens [7]. This results in a membrane structure devoid of cytoplasmic contents, yet retaining the original bacterial surface molecular topology, which underpins its excellent immunogenicity [8,9]. A key immunological advantage of BGs lies in their surface, which is rich in highly conserved pathogen-associated molecular patterns (PAMPs). These include peptidoglycan (PGN), lipoprotein (LPP), lipopolysaccharide (LPS), flagellin, fimbriae, and adhesins. These PAMPs function as natural immunostimulants, potently activating innate immune pathways and thereby conferring a self-adjuvant effect to BG vaccines [10,11,12,13]. Furthermore, the unique hollow cavity structure of BGs endows them with a significant secondary function as efficient delivery carriers for other antigens or therapeutic molecules [14,15,16].

Two primary strategies are employed for BG production: genetic engineering and chemical induction. The genetic approach typically hinges on the controlled expression of specific lysis genes, such as the phage PhiX174 gene E. The encoded protein forms transmembrane tunnels with diameters of 40–200 nm at sites bridging the inner and outer membranes, inducing cell lysis via osmotic imbalance [10]. Despite its conceptual elegance, this method is constrained by several practical limitations, including host-strain specificity, plasmid instability, frequently insufficient lysis efficiency, a relatively long preparation time, and high cost [17]. Moreover, effective lysis via genetic induction often requires the use of low bacterial concentrations, which hinders the demands of large-scale vaccine production. In contrast, chemical induction has emerged as a robust, versatile, and increasingly mainstream alternative [18,19]. This method utilizes chemical agents such as strong acids (e.g., HCl, H_2_SO_4_, HNO_3_) or bases (e.g., NaOH) at their minimum inhibitory concentration, often in combination with surfactants like Triton X-100, Tween-80, or sodium dodecyl sulfate (SDS), to disrupt membrane integrity under controlled conditions [20,21,22,23]. The integration of surfactants into multi-component reagent systems has proven particularly effective for successful BG generation [20,21,22,24]. While harsh reagents may risk damaging surface antigens, optimized protocols employing milder conditions or protective agents can effectively preserve immunogenicity. This practicality, scalability, and efficiency solidifies chemical induction as a highly promising approach for vaccine development, offering a solution to the bottlenecks associated with genetic methods.

Based on our preliminary work which established a chemical induction method for BGs preparation using sodium hydroxide, this study seeks to further optimize the process. Nonylphenol polyethoxylate-40 (NP-40), a nonionic surfactant widely used in biological research, has been demonstrated to effectively solubilize cell membranes without causing extensive protein denaturation. It is commonly employed to extract soluble proteins and solubilize membrane-associated proteins while preserving their structural and functional integrity. Therefore, in the present study, we aim to investigate whether the combined use of NP-40 and NaOH can further enhance the immunogenicity of *V. harveyi* ghosts as vaccine antigens. This work is expected to provide a theoretical foundation and a practical strategy for developing chemically induced bacterial ghost vaccines with improved immunogenic performance.

## 2. Methods and Materials

### 2.1. Fish Maintenance

Healthy turbot with an average body weight of 35.0 ± 5.0 g were sourced from a commercial aquaculture facility (Shandong, China). The fish were reared in aerated tanks supplied with continuously flowing sand-filtered seawater maintained at 15.0 ± 1.0 °C. During the acclimation period of at least one week prior to experimentation, the fish were fed twice daily with a commercial pellet diet. Prior to any experimental procedures, fish were anesthetized using MS-222 (100 ng/mL) dissolved in seawater. All animal experiments were conducted in compliance with the institutional guidelines and were approved by the Animal Research and Ethics Committees of East China University of Science and Technology (University Science and Technology Document ECUST-2026-001,31 December 2025).

### 2.2. Bacterial Strain

*V. harveyi* strain used in this study was originally isolated from diseased turbot in a Shandong mariculture farm. The strain was streaked on tryptic soy agar (TSA, Shanghai, China) and incubated at 28 °C for 24 h. A single colony from the plate was then inoculated into tryptic soy broth (TSB, Shanghai, China) and grown at 28 °C with shaking at 200 rpm for 12 h to serve as the primary seed culture. This primary culture was then transferred to a larger volume of fresh TSB and grown under the same conditions for another 12 h to obtain the secondary culture. For long term storage, the strain was kept at −80 °C in TSB containing 20% (*v*/*v*) glycerol.

### 2.3. Minimum Inhibitory Concentration (MIC) of Chemical Reagents

The secondary culture of *V. harveyi* was harvested and adjusted to a concentration of 1.0 × 10^9^ CFU/mL. To determine the MIC, the bacterial suspensions were treated with final concentrations of 0% or 1% (*v*/*v*) NP-40 (Beijing, China) and incubated at 28 °C with shaking at 200 rpm for 12 h. Following this pretreatment, the cultures were further exposed to NaOH at a highest final concentration of 5.8 mg/mL, followed by a series of two-fold serial dilutions, and incubated at 28 °C with shaking at 200 rpm. At predetermined time intervals, samples were collected, spread onto TSA plates, and incubated at 28 °C for 48 h. The MIC was defined as the lowest concentration of the NP-40/NaOH co-treatment that resulted in no visible colony growth, indicating complete bacterial inactivation.

### 2.4. Quality Assessment of BGs

#### 2.4.1. Morphological Examination by Scanning Electron Microscopy

To visualize the structural integrity and surface morphology of the prepared BGs, scanning electron microscopy was performed on three sample groups: untreated *V. harveyi* cells (VH), those treated with 2.90 mg/mL NaOH alone for 30 min (VHG_NaOH_), and those treated with 1% NP-40 and 1.45 mg/mL NaOH combination for 30 min (VHG_NP-40/NaOH_). All samples were adjusted to a uniform concentration of 1.0 × 10^9^ CFU/mL prior to chemical treatment. The samples were fixed with 2.5% glutaraldehyde at 4 °C overnight, washed with phosphate-buffered saline (PBS), and dehydrated through a graded ethanol series (30%, 50%, 70%, 90%, and 100%). After freeze-drying, the samples were sputter-coated with gold and observed under a scanning electron microscope S-4800 (Hitachi, Tokyo, Japan).

#### 2.4.2. Analysis of Cytoplasmic Content Removal

The efficiency of cytoplasmic content removal was assessed by examining the residual nucleic acid and protein in the prepared BGs. DNA retention was evaluated by extracting genomic DNA from untreated VH, VHG_NaOH_, and VHG_NP-40/NaOH_ using a commercial DNA extraction kit. The extracted DNA was mixed with 10× loading buffer and analyzed by agarose gel electrophoresis to visualize potential DNA residues. For protein analysis, bacterial suspensions of VH, VHG_NaOH_, and VHG_NP-40/NaOH_ were centrifuged at 8000 rpm for 10 min to separate cell associated fractions (precipitates) from released components (supernatants). All fractions were denatured with 5× protein loading buffer, boiled for 10 min, and subjected to SDS-PAGE. The gels were stained with Coomassie Brilliant Blue to visualize protein patterns and assess the extent of protein release from the bacterial structures.

#### 2.4.3. Evaluation of Lipopolysaccharide Integrity

The structural integrity of lipopolysaccharide (LPS) in the BGs was evaluated and compared with that of untreated *V. harveyi*. LPS was extracted from VH, VHG_NaOH_, and VHG_NP-40/NaOH_ samples using a commercial LPS extraction kit according to the manufacturer’s instructions. For electrophoresis analysis, the extracted LPS samples were pretreated by mixing with an equal volume of sample buffer containing β-mercaptoethanol, followed by boiling for 5 min. The denatured LPS samples were then separated on a 12.5% polyacrylamide gel using SDS-PAGE. After electrophoresis, the gels were specifically stained with a silver staining kit to visualize the LPS banding patterns, allowing for comparison of LPS structural preservation among the different treatment groups.

### 2.5. Vaccine Preparation

To compare the immunogenicity of chemically induced BGs against a conventional formaldehyde killed cells (FKC) as vaccines, three antigen types including VHG_NaOH_, VHG_NP-40/NaOH_ and FKC were prepared. Bacterial ghost vaccines were prepared from secondary cultures adjusted to 1.0 × 10^9^ CFU/mL. VHG_NaOH_ was generated by treatment with 2.90 mg/mL NaOH at 28 °C for 30 min, while VHG_NP-40/NaOH_ received sequential treatment with 1% NP-40 for 12 h followed by 1.45 mg/mL NaOH for 30 min. For FKC preparation, bacterial cultures were inactivated with 0.2% formaldehyde at 28 °C for 24 h. All preparations were confirmed to be completely inactivated by absence of bacterial growth on TSA plates after 3-day incubation, and vaccine antigens were adjusted to 5.0 × 10^9^ CFU/mL with sterile PBS.

### 2.6. Immunoprotective Efficacy Evaluation of Vaccines in Turbot

The immunoprotective efficacy of the prepared vaccines was evaluated in turbot. Fish were randomly divided into four groups with fifty fish per group. Three experimental groups received intraperitoneal injections of 100 μL containing VHG_NaOH_, VHG_NP-40/NaOH_ and FKC vaccines, respectively, while the control group received 100 μL of PBS.

#### 2.6.1. Protective Efficacy Assessment

At 28 days post vaccination (d.p.v), at least 30 fish were randomly selected from each group and challenged by intramuscular injection with 0.1 mL of *V. harveyi* containing 1.0 × 10^7^ CFU per fish. Mortality was recorded daily following challenge, and the relative percentage survival (RPS) was calculated using the formula:

$$\mathrm{RPS}\;=\left(1-\frac{\mathrm{mortality}\;\mathrm{of}\;\mathrm{vaccinated}\;\mathrm{fish}}{\mathrm{mortality}\;\mathrm{of}\;\mathrm{control}\;\mathrm{fish}}\right)\times \;100\%$$

#### 2.6.2. Specific Antibody Evaluation

To monitor the humoral immune response, serum samples were collected at 7, 14, 21, and 28 d.p.v. At each time point, three fish per group were euthanized with MS-222. Blood was drawn from the caudal vein and allowed to clot at 4 °C for 4 h, followed by centrifugation at 1500× *g* for 10 min at 4 °C to obtain serum. Serum antibody levels against *V. harveyi* were measured by enzyme linked immunosorbent assay (ELISA). High binding microplates were coated with 100 μL/well of *V. harveyi* suspension (1.0 × 10^8^ CFU/mL in PBS) and incubated overnight at 4 °C. After washing with PBST (PBS containing 0.05% Tween 20), plates were blocked with PBST containing 1% bovine serum albumin (BSA). Serial dilutions of turbot serum were added and incubated for 3 h at room temperature. Following washing, mouse anti-turbot IgM (Aquatic Diagnostics Ltd., Scotland, UK, 1:33 diluted by PBSTA) was added and incubated for 1 h. Plates were then incubated with horseradish peroxidase (HRP)-conjugated goat anti-mouse IgG (Abgent, San Diego, CA, USA, 1:200 diluted by PBSTA) for 1 h. Finally, the microplates were washed five times and 100 μL/well Tetramethylbenzidine (TMB) was used for color development at room temperature for 10 min in dark place, which was stopped by 50 μL/well H_2_SO_4_ (2 M). Optical density (OD) was read at 450 nm by microplates reader.

#### 2.6.3. Lysozyme Activity and Total Serum Protein

Lysozyme activity in turbot serum was determined using a turbidimetric assay. Briefly, 10 µL of serum was mixed with 10 µL of *Micrococcus lysodeikticus* suspension (OD_570_ = 0.3~0.5) in a pre-cooled 96-well microplate, with PBS serving as the negative control. The plate was immediately transferred to a microplate reader preheated to 28 °C, and the absorbance at 570 nm was recorded at 2 min intervals. Lysozyme activity was expressed as the slope of the absorbance-time curve.

Total serum protein content was quantified using a BCA protein assay kit (Beyotime, Shanghai, China) according to the manufacturer’s instructions. The BCA working solution was prepared by mixing reagents A and B at a 50:1 ratio. Subsequently, 20 µL of standard or diluted serum sample (1:128 dilution) was mixed with the working solution in a 96-well plate and incubated at 37 °C for 30 min. Absorbance was measured at 562 nm using a microplate reader.

### 2.7. Statistical Analysis

Survival rates after challenge were analyzed using the Kaplan–Meier method, and the overall differences among the four groups were compared with the log-rank (Man-tel-Cox) test. If a significant difference was detected, pairwise comparisons were conducted with Bonferroni correction for multiple comparisons. A *p*-value of less than 0.05 was considered statistically significant.

The other statistical analysis was performed by Graphpad Prism 9 or IBM SPSS 22 software. The statistical significance was analyzed by a two-way ANOVA. The data was presented as mean ± SD. Differences were considered significant at * *p* < 0.05, ** *p* < 0.01, and *** *p* < 0.001.

## 3. Results

### 3.1. Optimization of Bacterial Ghost Preparation Conditions

The MIC of the chemical inducers was determined to establish the optimal conditions for *V. harveyi* ghost preparation. As was shown in Table 1, treatment with NaOH alone required a concentration of 2.90 mg/mL for 30 min to achieve complete bacterial inactivation (VHG_NaOH_). However, when the bacterial suspension was pretreated with 1% NP-40 for 12 h prior to NaOH exposure (VHG_NP-40/NaOH_), the MIC of NaOH was reduced by half to 1.45 mg/mL for the same incubation period. This marked reduction in the required NaOH concentration demonstrates a clear synergistic effect between NP-40 and NaOH in inducing bacterial lysis, suggesting that the surfactant pretreatment enhances the susceptibility of *V. harveyi* to alkaline lysis.

### 3.2. Morphological Characterization of BGs

**Figure 1:**
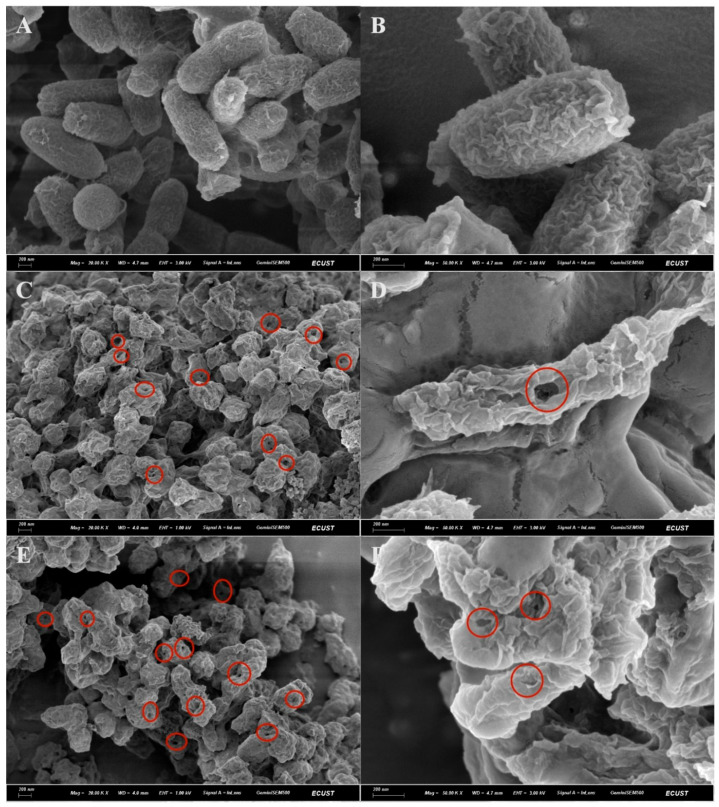
Morphological examination of *V. harveyi* and BGs by scanning electron microscopy. (**A**,**B**) Untreated VH cells. (**C**,**D**) VHG_NaOH_ treated cells. (**E**,**F**) VHG_NP-40/NaOH_ treated cells. The scale bar is indicated in the images. The obvious pores on the surface of the bacteria have been marked.

The surface structural integrity of the prepared BGs was assessed using scanning electron microscopy. As shown in Figure 1, untreated *V. harveyi* cells displayed intact surfaces with typical short-rod morphology. In contrast, both NaOH-treated (VHG_NaOH_) and NP-40/NaOH-treated bacteria (VHG_NP-40/NaOH_) exhibited apparent transmembrane pores and cellular collapse, indicative of substantial cytoplasmic content release. Despite these alterations, the overall cellular contours and surface architecture remained discernible in all ghost preparations. These results confirm that the chemical induction protocols successfully generated BGs through controlled lysis while preserving structural frameworks essential for vaccine development.

### 3.3. Analysis of Cytoplasmic Content Removal

**Figure 2:**
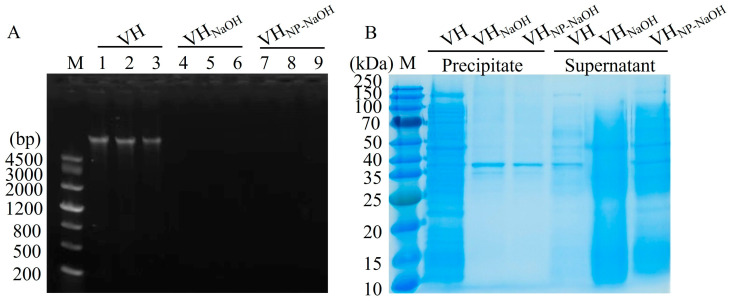
Analysis of cytoplasmic content removal in *V. harveyi* BGs. (**A**) Agarose gel electrophoresis of genomic DNA from VH (lanes 1–3), VHG_NaOH_ (lanes 4–6), and VHG_NP-40/NaOH_ (lanes 7–9). (**B**) SDS-PAGE analysis of protein distribution in precipitate (lanes 1–3: VH, VHG_NaOH_, VHG_NP-40/NaOH_) and supernatant (lanes 4–6: VH, VHG_NaOH_, VHG_NP-40/NaOH_) fractions.

To evaluate the efficiency of cytoplasmic content removal during ghost preparation, residual nucleic acids and proteins in the bacterial ghost samples were analyzed. Genomic DNA was extracted from untreated *V. harveyi* (VH), NaOH-induced ghosts (VHG_NaOH_), and NP-40/NaOH-induced ghosts (VHG_NP-40/NaOH_), followed by agarose gel electrophoresis. As illustrated in Figure 2A, a distinct DNA band was observed in the VH group (lanes 1–3), whereas no visible bands were detected in either the VHG_NaOH_ (lanes 4–6) or VHG_NP-40/NaOH_ groups (lanes 7–9). Quantitative analysis further revealed that the DNA concentrations in VH, VHG_NaOH_, and VHG_NP-40/NaOH_ were approximately 396.0 ng/µL, 10.5 ng/µL, and 9.4 ng/µL, respectively. These results indicate that both NaOH and NP-40/NaOH treatments effectively facilitated the efflux of intracellular DNA.

Protein distribution in the cell-associated (precipitate) and released (supernatant) fractions was assessed. As shown in Figure 2B, the precipitate of untreated VH (lanes 1) exhibited abundant protein bands. In contrast, the precipitates of VHG_NaOH_ (lanes 2) and VHG_NP-40/NaOH_ (lanes 3) displayed markedly reduced protein content, with only a faint band, presumably corresponding to membrane-associated proteins, remaining visible. In the supernatant fractions, minimal protein was detected in the VH (lanes 4) group, whereas both ghost preparations (lanes 5 and lanes 6) showed a diverse array of protein bands across a range of molecular weights, with the NP-40/NaOH combination exhibiting a marginally enhanced effect on protein clearance compared to NaOH treatment alone. These findings confirm that chemical induction using NaOH or NP-40/NaOH effectively promotes the release of cytoplasmic proteins.

### 3.4. LPS Integrity of BGs

**Figure 3:**
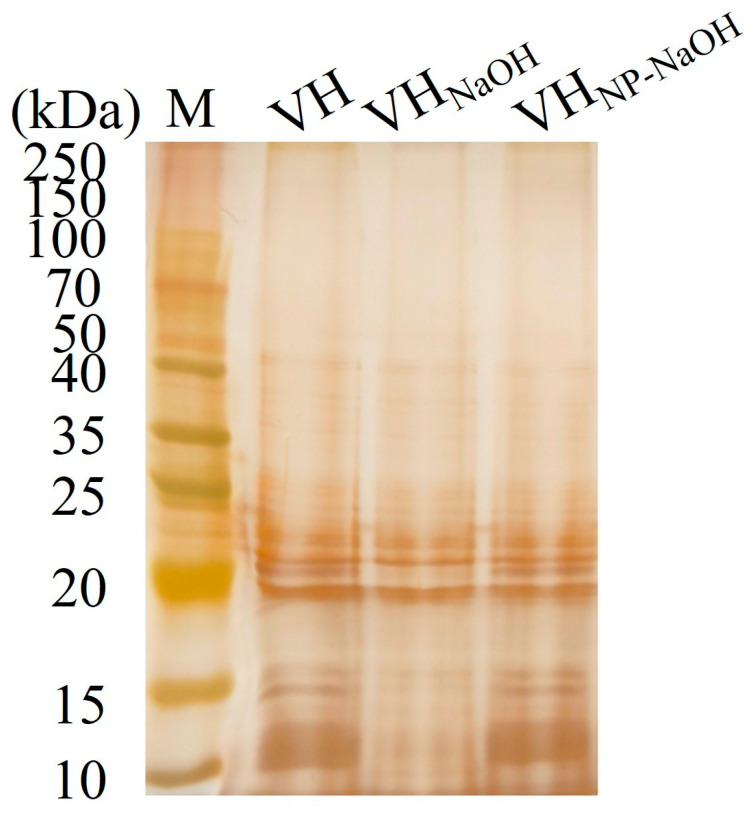
Analysis of lipopolysaccharide (LPS) integrity in *V. harveyi* bacterial ghosts. Silver-stained SDS-PAGE of LPS extracted from VH (lane 1), VHG_NaOH_ (lane 2), and VHG_NP-40/NaOH_ (lane 3).

The LPS profiles of the BGs preparations were analyzed and compared to those of untreated *V. harveyi*. As shown in Figure 3, the characteristic LPS ladder pattern was observed in the untreated VH group (lanes 1). In the VHG_NaOH_ group (lanes 2), this pattern showed minor band losses, indicating partial structural damage to the LPS. In contrast, the LPS banding profile of the VHG_NP-40/NaOH_ group (lanes 3) was largely preserved and closely resembled that of the untreated bacteria. These results demonstrate that the NP-40/NaOH combination better maintains LPS structural integrity compared to NaOH treatment alone.

### 3.5. Protective Efficacy of VHG

**Figure 4:**
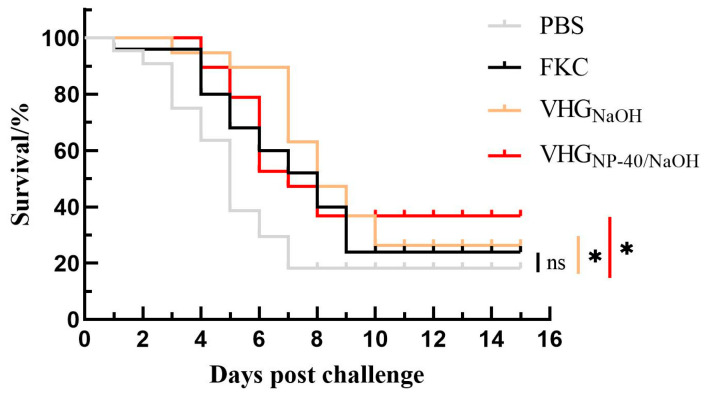
Kaplan–Meier survival curve of vaccinated turbot against a lethal challenge with *V. harveyi*. Cumulative survival count of fish immunized with PBS, FKC, VHG_NaOH_, or VHG_NP-40/NaOH_ following intramuscular challenge at 28 d.p.v. ns *p* > 0.05, * *p* < 0.05.

Based on the quality assessment of the BGs, the protective efficacy of vaccines prepared by chemical induction and conventional formaldehyde inactivation was evaluated in turbot. Fish were immunized with the respective vaccines and challenged with live *V. harveyi* at 28 d.p.v. As shown in Table 2 and Figure 4, the control group exhibited a cumulative mortality of 97.1%, whereas vaccination significantly reduced mortality across all vaccine groups. The mortality rates in fish immunized with FKC, VHG_NaOH_, and VHG_NP-40/NaOH_ were 63.3%, 43.8%, and 40.0%, respectively, corresponding to RPS values of 34.8%, 55.0%, and 58.8%. These results indicate that BGs elicit a more protective immune response compared to FKC, with the NP-40/NaOH combination conferring a modest but discernible enhancement in vaccine efficacy relative to treatment with NaOH alone.

### 3.6. Serum Specific IgM Antibody Levels

**Figure 5:**
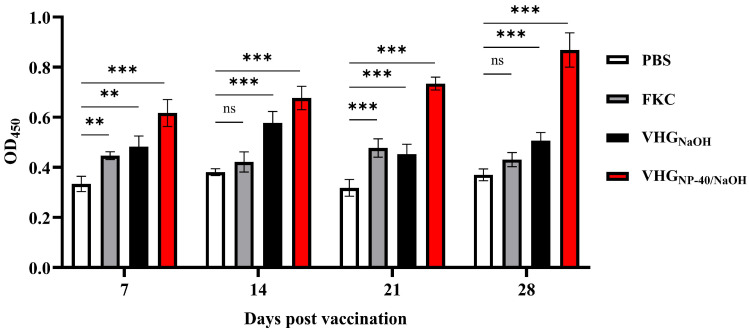
Serum-specific IgM antibody levels in vaccinated turbot. Serum was collected from fish immunized with PBS, FKC, VHG_NaOH_, or VH_GNP-40/NaOH_ at 7, 14, 21, and 28 d.p.v. Specific antibody levels against *V. harveyi* were measured by ELISA. Data are presented as mean ± SEM (*n* = 3). Significance was indicated as: ns *p* > 0.05, ** *p* < 0.01 and *** *p* < 0.001, vaccinated group vs. control group.

The humoral immune response elicited by vaccination was evaluated by measuring serum levels of specific IgM antibodies at 7, 14, 21, and 28 d.p.v. As shown in Figure 5, both the VHG_NaOH_ and VHG_NP-40/NaOH_ groups exhibited a significant increase in specific IgM titers from 7 d.p.v. onwards compared with FKC group. Moreover, the VHG_NP-40/NaOH_ group consistently induced significantly higher IgM levels than both the FKC and VHG_NaOH_ groups over the course of immunization, indicating a stronger and more sustained antibody response elicited by the NP-40/NaOH-induced BGs.

### 3.7. Lysozyme Activity and Total Serum Protein

**Figure 6:**
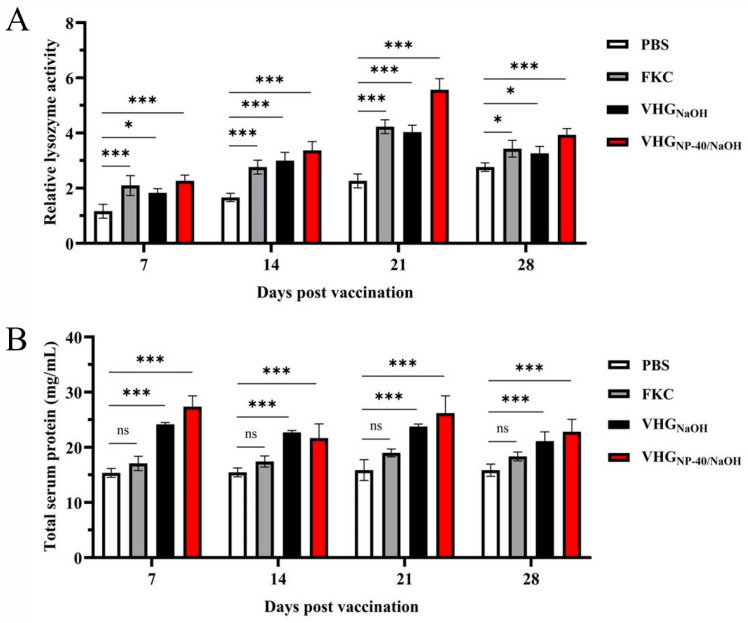
Analysis of serum lysozyme activity and total protein levels in vaccinated turbot. (**A**) lysozyme activity and (**B**) total protein concentration in serum collected from fish immunized with PBS, FKC, VHG_NaOH_, or VHG_NP-40/NaOH_ at 7, 14, 21, and 28 d.p.v. Lysozyme activity was determined by a turbidimetric assay, and total protein concentration was quantified using a BCA assay. Significance was indicated as: ns *p* > 0.05, * *p* < 0.05 and *** *p* < 0.001, vaccinated group vs. control group.

To assess the activation of innate immunity, serum lysozyme activity was monitored post-vaccination. As shown in Figure 6A, all vaccine groups exhibited elevated lysozyme activity compared to the control group, with levels increasing progressively and peaking at 21 d.p.v. At this time point, lysozyme activities reached 4.2 U, 4.0 U, and 5.6 U for the FKC, VHG_NaOH_, and VHG_NP-40/NaOH_ groups, respectively. These results indicate that all tested vaccines could activate the innate immune response in turbot.

Serum total protein levels were also measured to further evaluate the humoral immune status. As presented in Figure 6B, fish immunized with VHG_NaOH_ and VHG_NP-40/NaOH_ showed a rapid increase in total protein, reaching peak levels as early as 7 d.p.v., with the VHG_NP-40/NaOH_ group achieving a concentration of approximately 27.4 mg/mL. Furthermore, throughout the entire observation period, total protein levels in both bacterial ghost groups were higher than those in the FKC group. This pattern suggests a potent and sustained activation of antibody mediated humoral immunity by the ghost vaccines, particularly those prepared with the NP-40/NaOH combination.

## 4. Discussion

BGs, as empty bacterial cell envelopes retaining native surface structures, represent a promising vaccine platform by preserving PAMPs while eliminating replicative risks. Building upon our preliminary work that established NaOH-induced lysis for ghost preparation [25], this study introduced NP-40, a nonionic surfactant known for its mild membrane solubilizing properties and capacity to maintain protein integrity, to further optimize the process. While NP-40 is widely used in protein extraction buffers, its application in BGs production had not been previously reported. Our findings demonstrate that combining NP-40 with NaOH not only enhances lysis efficiency but also better preserves critical surface antigens, leading to improved immunogenicity of the resulting ghost vaccines.

Presently, the use of NaOH for BG preparation is well-documented, as demonstrated by its successful application in generating *Staphylococcus aureus* ghosts [26,27] and its identification as the optimal choice for *Vibrio parahaemolyticus* ghost vaccines, a finding consistent with our prior work on *Aeromonas salmonicida* [25]. In the present study, we confirmed that NaOH alone at 2.90 mg/mL was sufficient for complete inactivation of *V. harveyi*. Notably, pretreatment with 1% NP-40 reduced the MIC of NaOH by half to 1.45 mg/mL, revealing a clear synergistic lysis effect. This strategy of using multi-component chemical systems aligns with the pioneering work of Amara et al. [18,28]. This concept finds further support in a study by Abdelfattah et al., where a 12 h pretreatment with Triton X-100 significantly reduced the required concentration of lactic acid for generating *Shigella flexneri* ghosts, enhancing the release of nucleic acids and proteins [20]. The observed enhancement suggests that NP-40 pretreatment effectively permeabilizes the bacterial membrane, thereby increasing its susceptibility to alkaline lysis and enabling efficient ghost formation under milder conditions.

The integrity of surface structures and the efficient removal of cytoplasmic components are critical criteria for evaluating bacterial ghost quality. Extensive expulsion of nucleic acids and proteins ensures biological safety by eliminating genetic replication risks, while well-preserved surface architecture, particularly LPS, is essential for retaining conformational antigenic epitopes and thus vaccine immunogenicity [11]. Our quality assessments confirmed that both NaOH and NP-40/NaOH treatments successfully generated structurally intact ghosts with visible transmembrane pores and effective cytoplasmic content expulsion. However, comparative analysis revealed distinct advantages of the combined approach. While cytoplasmic clearance was efficient in both preparations, LPS integrity, a key immunogen, was significantly better preserved in VHG_NP-40/NaOH_. The superior structural preservation achieved with our NP-40/NaOH combination likely stems from the membrane-stabilizing properties of the nonionic surfactant, which mitigates the collateral damage typically associated with alkaline lysis.

As an inactivated vaccine platform, chemically induced BGs offer superior biosafety compared to genetic methods, while overcoming the critical limitation of traditional formaldehyde inactivation. To directly compare immunogenicity without confounding adjuvant effects, we evaluated FKC and BGs in unadjuvanted formulations. The superior antigenic preservation achieved with NP-40/NaOH treatment translated directly into enhanced protective efficacy, with VHG_NP-40/NaOH_ providing the highest protection (RPS = 58.8%).

The immunologic mechanisms underlying this protection were elucidated through serological analyses. Specific IgM antibodies serve as crucial markers of adaptive humoral immunity activation. In our study, the significantly higher and sustained IgM levels in the VHG_NP-40/NaOH_ group indicate robust B-cell activation and antibody production, directly correlated with the observed protective efficacy. This enhanced humoral response was further supported by total serum protein measurements, where elevated levels particularly in ghost vaccine groups reflect increased globulin synthesis, consistent with established correlations between total protein content and antibody-mediated immunity in teleost fish [29]. Simultaneously, lysozyme activity, a key indicator of innate immune function primarily secreted by macrophages and neutrophils, showed marked elevation in all vaccine groups. The highest lysozyme activity in VHG_NP-40/NaOH_ vaccinated fish demonstrates potent activation of non-specific defense mechanisms, which is critical for early pathogen clearance [30]. This coordinated enhancement of both innate (elevated lysozyme) and adaptive (increased IgM and total protein) immune responses underscores the advantage of BGs in providing comprehensive immune stimulation.

However, despite the superior immune parameters observed in the VHG_NP-40/NaOH_ group, the marginal difference in RPS compared to VHG_NaOH_ suggests that the protective efficacy of bacterial ghost vaccines may reach a plateau once critical antigenic structures are adequately preserved. Both ghost preparations likely provided sufficient PAMPs and antigens to activate essential immune pathways required for protection, creating a threshold effect where further immunogenicity improvements yield diminishing returns in survival rates. This phenomenon indicates that while NP-40/NaOH optimization enhances immune activation, the NaOH-alone method already achieves substantial protection, and the added benefit may be more pronounced under different challenge conditions or in long-term immunity.

## 5. Conclusions

In summary, this study establishes an optimized NP-40/NaOH protocol for *V. harveyi* ghost preparation that demonstrates dual advantages: enhanced lysis efficiency and superior antigen preservation. The resulting BGs elicited robust innate and adaptive immune responses in turbot, providing the highest protection against vibriosis. This work validates the NP-40/NaOH combination as an effective strategy for developing chemically induced bacterial ghost vaccines, with promising applications in aquaculture disease prevention.

## Figures and Tables

**Table 1:** Determination of minimal inhibitory concentration for *V. harveyi* ghost preparation.

NP-40 (*v*/*v*)	NaOH (mg/mL)				
	5.8000	2.9000	1.4500	0.7250	0.3625
0%	-	-	+	+	+
1%	-	-	-	+	+

“+” means VH growth, “-” means VH died.

**Table 2:** Relative percentage survival of vaccinated turbot after challenge.

Group	Totality	Mortality	Average Mortality (%)	RPS (%)
PBS	35	34	97.1%	/
FKC	30	19	63.3%	34.8%
VHG_NaOH_	32	14	43.8%	55.0%
VHG_NP-40/NaOH_	30	12	40.0%	58.8%

## Data Availability

All the data will be provided on reasonable request.
